# Magnesium-Deficiency Effects on Pigments, Photosynthesis and Photosynthetic Electron Transport of Leaves, and Nutrients of Leaf Blades and Veins in *Citrus sinensis* Seedlings

**DOI:** 10.3390/plants8100389

**Published:** 2019-09-30

**Authors:** Xin Ye, Xu-Feng Chen, Chong-Ling Deng, Lin-Tong Yang, Ning-Wei Lai, Jiu-Xin Guo, Li-Song Chen

**Affiliations:** 1 Institute of Plant Nutritional Physiology and Molecular Biology, College of Resources and Environment, Fujian Agriculture and Forestry University, Fuzhou 350002, China; yexin1000@fafu.edu.cn (X.Y.); 1190807003@fafu.edu.cn (X.-F.C.); talstoy@fafu.edu.cn (L.-T.Y.); lainingwei1109@fafu.cn (N.-W.L.); jxguo@fafu.edu.cn (J.-X.G.); 2 Guangxi Key Laboratory of Citrus Biology, Guangxi Academy of Specialty Crops, Guilin 541004, China; cldeng88168@126.com; 3 Fujian Provincial Key Laboratory of Soil Environmental Health and Regulation, College of Resources and Environment, Fujian Agriculture and Forestry University, Fuzhou 350002, China; 4 Key Lab of Soil Ecosystem Health and Regulation (Fujian Agriculture and Forestry University), Fujian Province University, Fuzhou 350002, China

**Keywords:** *Citrus sinensis*, leaf blade, magnesium (Mg)-deficiency, OJIP transient, photosynthesis, vein enlargement and corkiness

## Abstract

*Citrus sinensis* seedlings were irrigated with nutrient solution at a concentration of 0 (Mg-deficiency) or 2 (Mg-sufficiency) mM Mg (NO_3_)_2_ for 16 weeks. Mg-deficiency-induced interveinal chlorosis, vein enlargement and corkiness, and alterations of gas exchange, pigments, chlorophyll a fluorescence (OJIP) transients and related parameters were observed in middle and lower leaves, especially in the latter, but not in upper leaves. Mg-deficiency might impair the whole photosynthetic electron transport, including structural damage to thylakoids, ungrouping of photosystem II (PSII), inactivation of oxygen-evolving complex (OEC) and reaction centers (RCs), increased reduction of primary quinone electron acceptor (Q_A_) and plastoquinone pool at PSII acceptor side and oxidation of PSI end-electron acceptors, thus lowering energy transfer and absorption efficiency and the transfer of electrons to the dark reactions, hence, the rate of CO_2_ assimilation in Mg-deficiency middle and lower leaves. Although potassium, Mg, manganese and zinc concentration in blades displayed a significant and positive relationship with the corresponding element concentration in veins, respectively, great differences existed in Mg-deficiency-induced alterations of nutrient concentrations between leaf blades and veins. For example, Mg-deficiency increased boron level in the blades of upper leaves, decreased boron level in the blades of lower leaves, but did not affect boron level in the blades of middle leaves and veins of upper, middle and lower leaves. To conclude, Mg-deficiency-induced interveinal chlorosis, vein enlargement, and corkiness, and alterations to photosynthesis and related parameters increased with increasing leaf age. Mg-deficiency-induced enlargement and corkiness of veins were not caused by Mg-deficiency-induced boron-starvation.

## 1. Introduction

Magnesium (Mg), as a central element of the chlorophyll (Chl) molecule and the activator of more than 300 enzymes, plays key roles in various physiological and biochemical processes, including Chl biosynthesis and photosynthesis [1,2,3,4,5]. Despite its vital roles in the normal growth and development of higher plants, less attention has been paid to Mg in comparison to nitrogen (N), phosphorus (P), and potassium (K) [6]. Mg-deficiency, one of the most common physiological disorder influencing productivity and quality of crops including *Citrus*, is being intensified in intensive agricultural systems, where N, P, and K fertilizers have been heavily applied [7,8,9,10,11].

In addition to interveinal chlorosis, the decrease in leaf CO_2_ assimilation in response to Mg-deficiency is often observed in many higher plants, including *Citrus* [10,12,13,14,15,16]. As the transport of Mg from the old leaves to the young leaves is elevated under Mg-limited conditions, Mg-deficiency symptoms first appear in old leaves, and progressively extend toward the young leaves as the exposure duration of Mg-deficiency prolongs [1,3,8]. This leads us to hypothesize that Mg-deficiency-induced decreases in leaf pigments and photosynthesis, and alterations to Chl, fluorescence (OJIP) transients, and related fluorescence parameters might become more pronounced with increasing leaf age. To our knowledge, such data are very limited. Moreover, the results are not consistent. Li et al. and Cai et al. observed that CO_2_ assimilation, stomatal conductance (g_s_), and Chl a, Chl b, Chl a + b, and carotenoid (Car) concentrations were greatly decreased in Mg-deficiency lower leaves of *Citrus sinensis* seedlings, but unaltered in Mg-deficiency upper leaves except for a slight decrease in CO_2_ assimilation [8,11]. Although a few, very small necrotic spots occurred in source leaves of Mg-deficiency spinach leaves, Chl concentration in Mg-deficiency source and sink leaves remained unaltered relative to controls [17]. Kobayashi et al. investigated the effects of 6 and 8 days of Mg-deficiency on Chl, transpiration rate, and photosynthesis in leaf 4, leaf 5, and leaf 6 blades (from bottom to top) of rice seedlings. Chl level was reduced only in leaf 5 and leaf 6 on day 8. Transpiration in leaf 5 was 55% and 26% lower than that in controls on days 6 and 8, respectively [18]. Photosynthesis was decreased only in leaf 5 on day 8.

Monitoring OJIP has been widely used to examine the effects of nutrients (viz. Mg, N, P, K, calcium (Ca), sulfur (S), boron (B), iron (Fe), and manganese (Mn)) deficiencies on the behavior of the photosystem II (PSII) [19,20,21,22,23,24,25,26,27], since OJIP is a nonmultilative, super-sensitive, reliable, rapidly and simply measured approach, and it can provide a great deal of significant data about the photosynthetic apparatus [28,29,30,31]. However, very limited data are available on the effects of nutrient deficiencies on OJIP transients and related fluorescence parameters in different age (positioned) leaves. In a study with winter wheat, Živčák et al. found that N-deficiency lowered maximum PSII efficiency of dark-adapted leaves (F_v_/F_m_), performance index (PI_abs_), and total performance index (PI_abs,total_) in the first, youngest leaf (leaf 1) and the third leaf (leaf 3), especially in leaf 3 with the only exception that F_v_/F_m_ in the leaf 1 was not decreased by N deficiency [32]. Recently, Cai et al. observed Mg-deficiency-induced decreases in F_v_/F_m_, fraction of oxygen-evolving complex (OEC) in comparison with control and PI_abs,total_, and increases in quantum yield for energy dissipation (DI_o_/ABS) and dissipated energy flux per reaction center (RC, DI_o_/RC) were greater in lower leaves than those in upper leaves in *C. sinensis* seedlings [8]. Unfortunately, OJIP transients and many other fluorescence parameters were not provided in the two studies.

Magnesium-deficiency affects the uptake of nutrients and their concentrations in roots, stems, and leaves [1,2,33,34]. Evidence shows that Mg-deficiency may improve the concentrations of other cations, such as Ca, K, and Mn [2,11,35,36]. To our knowledge, very little is known about Mg-deficiency effects on concentrations of nutrients in various positioned (age) leaves. In a study with maize, Jezek et al. observed that Mg-deficiency increased the concentrations of K and Mg in the leaf 6, leaf 7, leaf 8, and the youngest leaf, and Mg-deficiency-induced alteration of K concentration was greater in the leaf 6 and leaf 7 than those in the leaf 8 and youngest leaf, while Mg-deficiency-induced alteration of Mn concentration was the lowest in the leaf 6 [2].

Leaf vein networks play key roles in transporting nutrition effectively into leaf cells [37,38]. The concentrations of some elements were higher in leaf veins than those in blades [38,39]. Thus, nutrient-deficiency-induced alterations of element levels might be different between leaf vein and blades. So far, such data have not been reported.

Like boron (B)-deficiency symptoms [40], enlargement and corkiness of midribs and main lateral veins often occur in Mg-deficiency *Citrus* leaves [8,12,33]. However, it is not clear whether the symptoms in Mg-deficiency leaves are caused by Mg-deficiency-induced B-starvation or not. So far, such symptoms were not found in other higher plants.

Here, we used *C. sinensis* seedlings as materials, and investigated Mg-deficiency symptoms and effects on pigments, gas exchange, OJIP transients, and related fluorescence parameters in upper, middle, and lower leaves, and concentrations of Mg and other elements in the blades (midribs, main lateral veins, and petioles removed) and veins of upper, middle, and lower leaves. The objectives were (a) to corroborate the hypotheses that Mg-deficiency-induced interveinal chlorosis, vein enlargement and corkiness, and alterations of photosynthesis and related physiological parameters increase with increasing leaf age and that Mg-deficiency-induced alterations to element levels differ between leaf blades and veins, and (b) to determine whether Mg-deficiency-induced enlargement and corkiness of veins are caused by Mg-deficiency-induced B-starvation.

## 2. Results

### 2.1. Mg-Deficiency Symptoms in C. sinensis Seedlings

**Figure 1:**
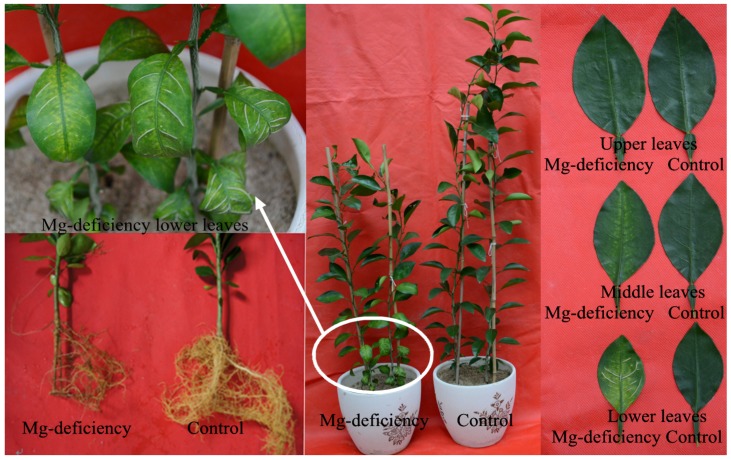
Mg-deficiency symptoms on *Citrus sinensis* seedlings.

As shown in Figure 1, 0 mM Mg treatment markedly inhibited root and shoot growth. Mg-deficiency symptoms first appeared in the basal older leaves, and then gradually extended toward the top young leaves. Interveinal chlorosis was observed in 0 mM Mg-treated middle and lower leaves, but not in 0 mM Mg-treated upper leaves. Enlargement and corkiness of midribs and main lateral veins were observed in a large number of 0 mM Mg-treated lower leaves and a small number of 0 mM Mg-treated middle leaves. No such Mg-deficiency symptoms occurred in 2 mM Mg-treated upper, middle, and lower leaves. Thus, seedlings that received 2 mM and 0 mM Mg were considered as Mg-sufficiency (control) and Mg-deficiency, respectively.

### 2.2. Leaf Pigments and Gas Exchange 

**Figure 2:**
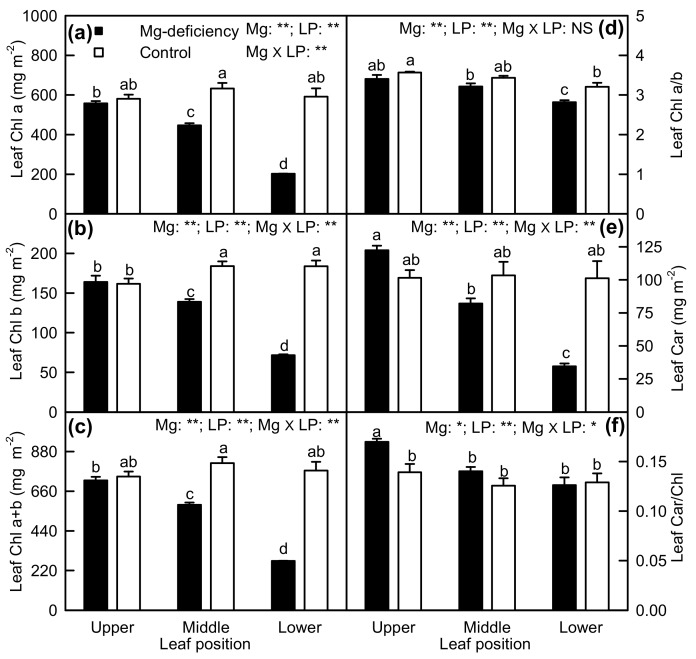
Mg-deficiency effects on Chl a (**a**), Chl b (**b**), Chl a + b (**c**), Chl a/b ratio (**d**), Car (**e**) and Car/Chl ratio (**f**) in *C. sinensis* leaves. Bars represent mean ± standard error (*n* = 4). Data were analyzed by two-way ANOVA (three (leaf positions) × two (Mg levels)) followed by Duncan's multiple range. Different letters above the bars indicate a significant difference at *p <* 0.05. NS, *, and ** indicate nonsignificant, significant at 5% and 1% level, respectively. LP, leaf position.

As shown in Figure 2, Mg-deficiency lowered Chl a, Chl b, Chl a + b, and Car concentrations in middle and lower leaves, but had no influence on their concentrations in upper leaves. Mg-deficiency decreased the Chl a/b ratio in lower leaves and increased the Car/Chl ratio in upper leaves. Under Mg-deficiency, Chl a, Chl b, Chl a + b, and Car concentrations decreased with increasing leaf age, but under Mg-sufficiency, their concentrations did not vary with leaf positions except for a slightly decreased Chl a concentration in upper leaves. The Chl a/b ratio slightly decreased with increasing leaf age, but the Car/Chl ratio was similar among the six treatment combinations except for a significant increase in Mg-deficiency upper leaves. There were significant interactions between Mg and leaf positions for the six parameters except for Chl a/b .

**Figure 3:**
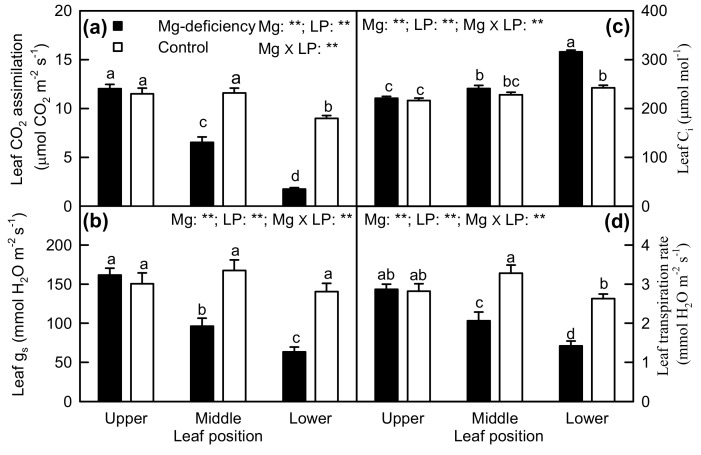
Mg-deficiency effects on CO_2_ assimilation (**a**), stomatal conductance (g_s_, (**b**)), intercellular CO_2_ concentration (C_i_, (**c**)), and transpiration rate (**d**) in *C. sinensis* leaves. Bars represent the mean ± standard error (*n* = 6). Data were analyzed by two-way ANOVA (three (leaf positions) × two (Mg levels)) followed by Duncan's multiple range. Different letters above the bars indicate a significant difference at *p <* 0.05. ** indicates a significant difference at *p* < 0.01. LP, leaf position.

Magnesium-deficiency decreased CO_2_ assimilation, g_s_, and transpiration rate in middle and lower leaves and increased intercellular CO_2_ concentration (C_i_) in lower leaves, but did not alter CO_2_ assimilation, g_s_, and transpiration rate in upper leaves and C_i_ in upper and middle leaves. Under Mg-deficiency, CO_2_ assimilation, g_s_, and transpiration rate decreased, but C_i_ increased with increasing leaf age. Under Mg-sufficiency, the four parameters displayed little change except for slightly decreased CO_2_ assimilation and transpiration rate and slightly increased C_i_ in lower leaves. Interactions between Mg and leaf positions were significant for the four parameters (Figure 3).

**Figure 4:**
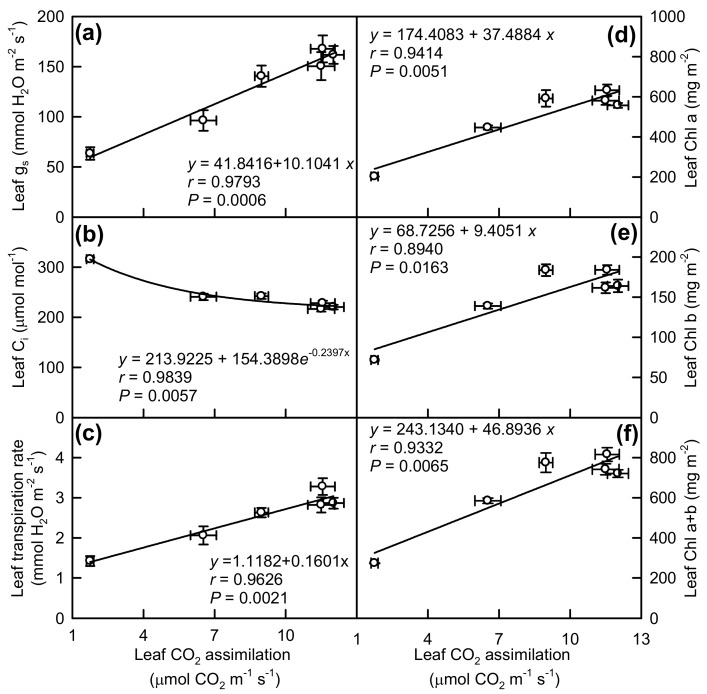
CO_2_ assimilation in relation to g_s_ (**a**), C_i_ (**b**), transpiration rate (**c**), Chl a (**d**), Chl b (**e**), and Chl a + b (**f**) in leaves. Point represents the mean ± standard error for the independent variable (*n* = 6) and the dependent variables (*n* = 4 or 6).

Leaf CO_2_ assimilation was positively related to g_s_, transpiration rate, Chl a, Chl b and Chl a + b concentration, respectively, but it was negatively related to C_i_ (Figure 4).

### 2.3. Leaf OJIP Transients and Related Parameters

**Figure 5:**
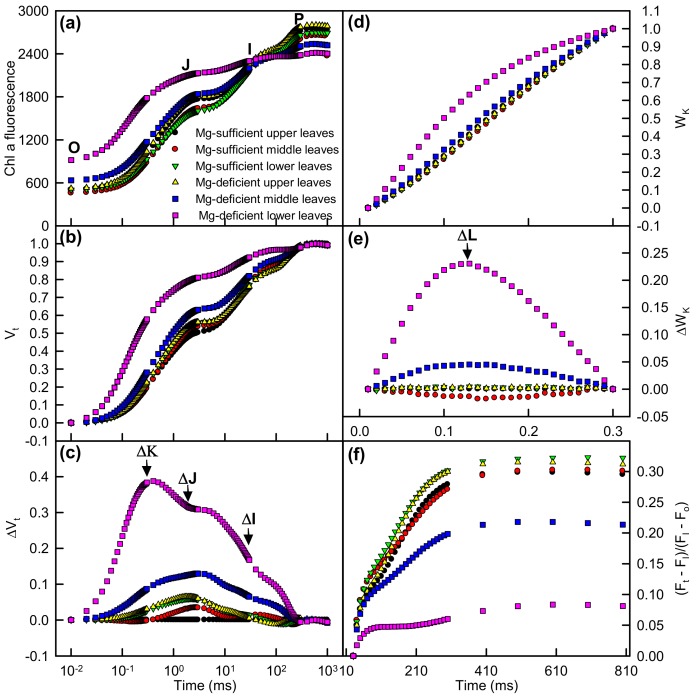
Mg-deficiency effects on the mean OJIP transients of four dark-adapted leaves (**a**) and the different expressions derived from the mean transients in dark-adapted leaves (**b**): between F_o_ and F_m_: V_t_ = (F_t_ − F_o_) / (F_m_ − F_o_), and (**c**) the differences of samples to the control sample (Mg-sufficient upper leaves); (**d**) between F_o_ and F_300µs_: W_k_ = (F_t_ − F_o_) / (F_300µs_ − F_o_) and (**e**) the differences of the samples to control sample, and (**f**) IP phase: (F_t_ − F_o_) / (F_I_ − F_o_) − 1 = (F_t_ − F_I_) / (F_I_ − F_o_).

Compared with Mg-sufficient upper leaves, Mg-deficiency middle and upper leaves had an increased O-step and a decreased P-step, especially in Mg-deficiency lower leaves. Similarly, positive ΔL-, ΔK-, ΔJ-, and ΔI-bands and decreased maximum amplitude of IP phase [(F_m_ − F_I_)/(F_I_ − F_o_)] were observed only in Mg-deficiency middle and lower leaves, especially in the latter. Under Mg-sufficiency, leaf positions had little influence on OJIP transients (Figure 5).

**Figure 6:**
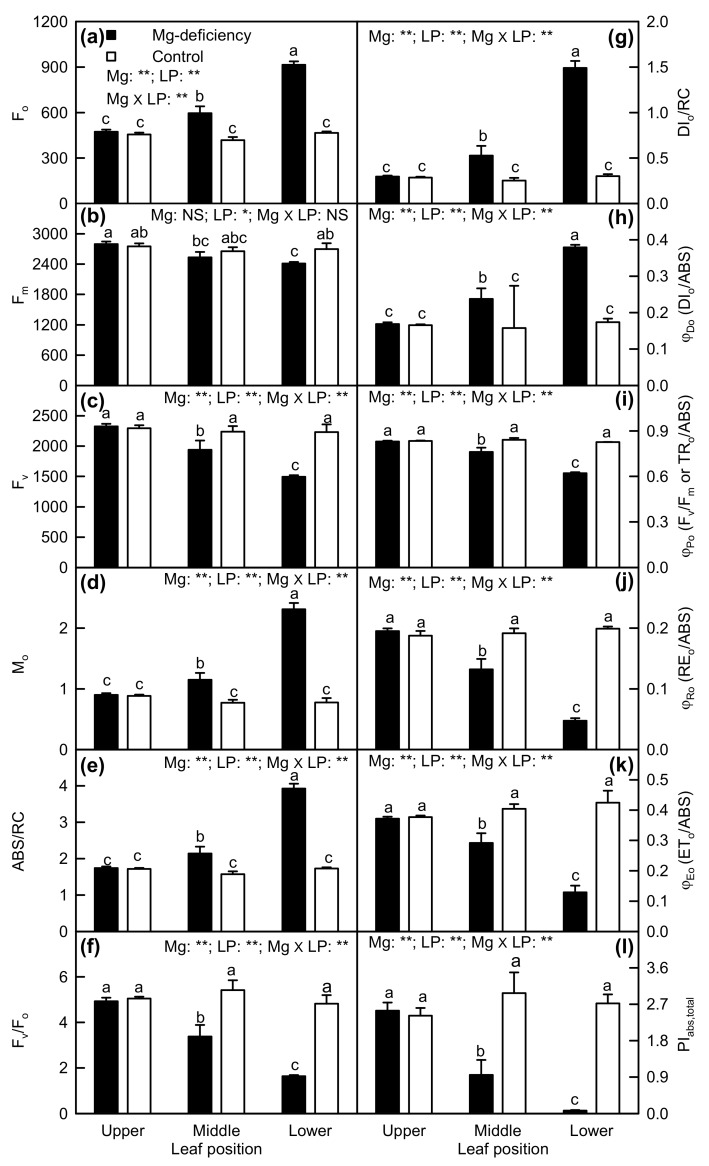
Mg-deficiency effects on F_o_ (**a**), F_m_ (**b**), F_v_ (**c**), M_o_ (**d**), ABS/RC (**e**), F_v_/F_o_ (**f**), DI_o_/RC (**g**), DI_o_/ABS (**h**), F_v_/F_m_ (**i**), RE_o_/ABS (**j**), ET_o_/ABS (**k**) and PI_abs,total_ (**l**) in *C. sinensis* leaves. Bars represent the mean ± standard error (*n* = 4). Data were analyzed by two-way ANOVA (three (leaf positions) × two (Mg levels)) followed by Duncan's multiple range. Different letters above the bars indicate a significant difference at *p <* 0.05. NS, *, and ** indicate nonsignificant, significant at 5% and 1% level, respectively. LP, leaf position.

Minimum fluorescence (F_o_), approximated initial slope (in ms^-1^) of the fluorescence transient V = f(t) (M_o_), absorption flux per RC (ABS/RC), DI_o_/RC, and DI_o_/ABS were increased only in Mg-deficiency middle and lower leaves, especially in the latter, but maximum fluorescence (F_m_), maximum variable fluorescence (F_v_), F_v_/F_m_, maximum primary yield of photochemistry of PSII (F_v_/F_o_), quantum yield of electron transport from the reduced primary quinone electron acceptor of PSII (Q_A_^-^) to the PSI end electron acceptors (RE_o_/ABS), quantum yield for electron transport (ET_o_/ABS) and PI_abs,total_ were decreased only in Mg-deficiency middle and lower leaves, especially in the latter. Under Mg-sufficiency, all these parameters were not significantly altered by leaf positions. Interactions between Mg and leaf positions were significant for all these parameters except for F_m_ (Figure 6). 

**Figure 7:**
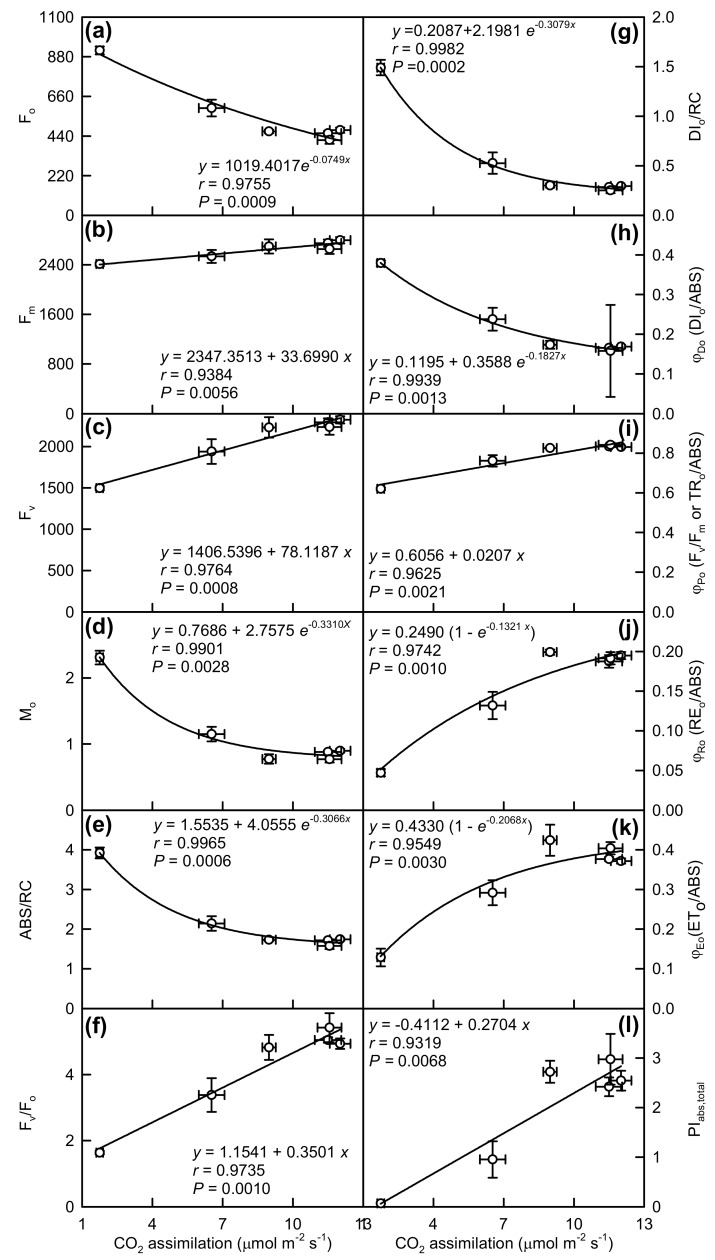
Leaf CO_2_ assimilation in relation to F_o_ (**a**), F_m_ (**b**), F_v_ (**c**), M_o_ (**d**), ABS/RC (**e**), F_v_/F_o_ (**f**), DI_o_/RC (**g**), DI_o_/ABS (**h**), F_v_/F_m_ (**i**), RE_o_/ABS (**j**), ET_o_/ABS (**k**) and PI_abs,total_ (**l**). Points represent means ± SE for the independent variable (*n* = 6) and the dependent variables (*n* = 4).

As shown in Figure 7, leaf CO_2_ assimilation was negatively related to F_o_, M_o_, ABS/RC, DI_o_/RC, and DI_o_/ABS, respectively, but it was positively related to F_m_, F_v_, F_v_/F_o_, F_v_/F_m_, RE_o_/ABS, ET_o_/ABS, and PI_abs,total_, respectively.

### 2.4. Element Concentrations in Leaf Blades and Veins

**Figure 8:**
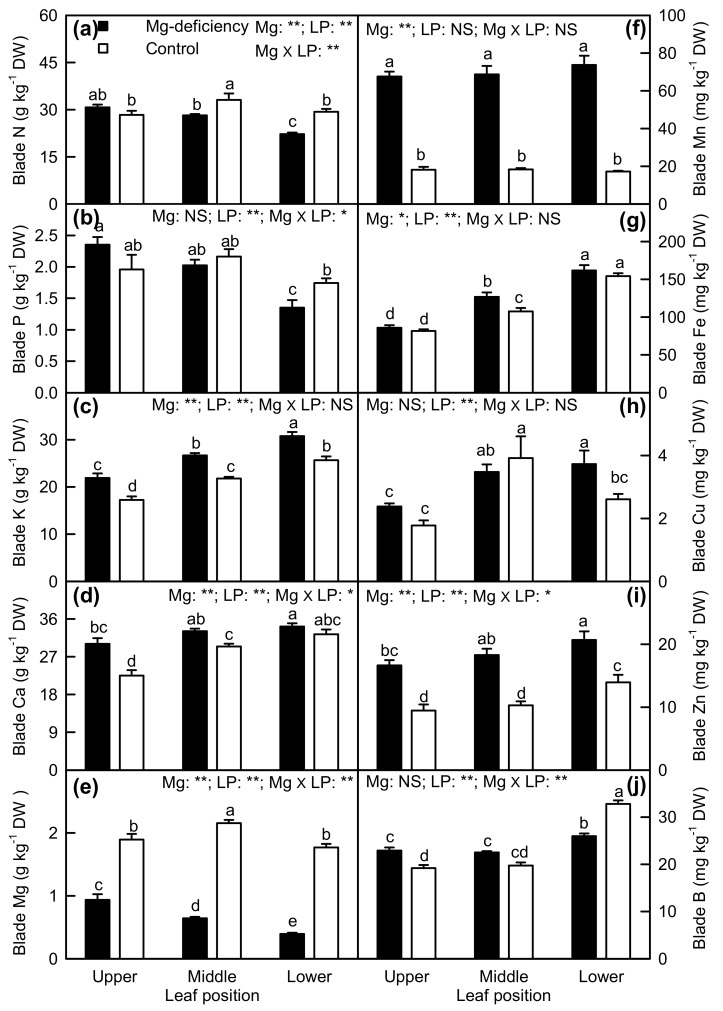
Mg-deficiency effects on the concentrations of N (**a**), P (**b**), K (**c**), Ca (**d**), Mg (**e**), Mn (**f**), Fe (**g**), Cu (**h**), Zn (**i**) and B (**j**) in the blades of upper, middle, and lower leaves. Bars represent the mean ± standard error (*n* = 4–6). Data were analyzed by two-way ANOVA (three (leaf positions) × two (Mg levels)) followed by Duncan's multiple range. Different letters above the bars indicate a significant difference at *p <* 0.05. NS, *, and ** indicate nonsignificant, significant at 5% and 1% level, respectively. LP, leaf position.

Mg-deficiency decreased or did not alter N, P, and Mg concentrations in the blades, but it increased or did not affect K, Ca, Mn, Fe, Cu, and Zn concentrations in the blades. B concentration was decreased in Mg-deficiency the blades of lower leaves, but it was increased in Mg-deficiency the blades of upper leaves. Under Mg-sufficiency, N, Mg, and Cu concentrations were significantly higher in the blades of middle leaves than those in the blades of upper and lower leaves; K, Ca, and Fe concentrations in leaf blades increased with increasing leaf age; Zn and B concentrations were higher in the blades of lower leaves than those in the blades of upper and middle leaves; P and Mn concentrations were similar among the blades of lower, middle, and upper leaves. Under Mg-deficiency, N, P, and Mg concentrations in the blades displayed a decreased trend with increasing leaf age; K, Ca, Fe, Cu, and Zn concentrations in the blades displayed an increasing trend with increasing leaf age; Mn concentration was similar among the blades of lower, middle and lower leaves; B concentration was higher in the blades of lower leave than that in the blades of upper and middle leaves. Interactions between Mg and leaf positions were significant only for the blade N, P, Ca, Mg, Zn, and B concentrations (Figure 8). 

**Figure 9:**
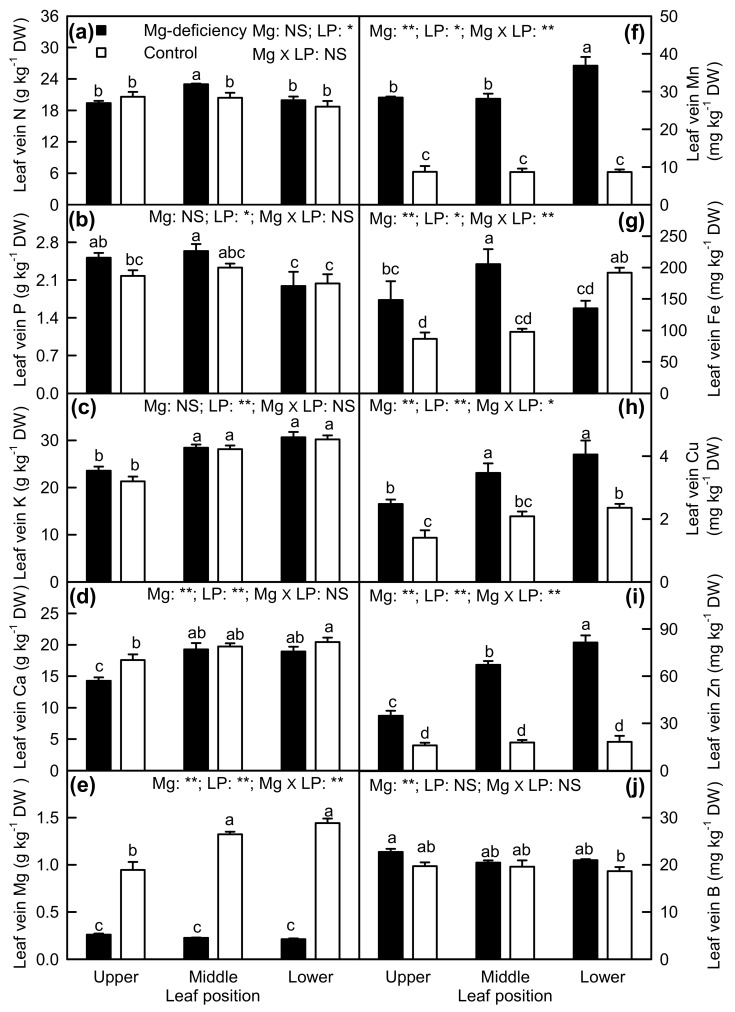
Mg-deficiency effects on the concentrations of N (**a**), P (**b**), K (**c**), Ca (**d**), Mg (**e**), Mn (**f**), Fe (**g**), Cu (**h**), Zn (**i**) and B (**j**) in the veins of upper, middle, and lower leaves. Bars represent the mean ± standard error (*n* = 4–6). Data were analyzed by two-way ANOVA (three (leaf positions) × two (Mg levels)) followed by Duncan's multiple range. Different letters above the bars indicate a significant difference at *p <* 0.05. NS, *, and ** indicate nonsignificant, significant at 5% and 1% level, respectively. LP, leaf position.

Magnesium-deficiency increased or had no significant influence on N, Mn, Cu, and Zn concentrations in veins, decreased or did not significantly alter Ca and Mg concentrations in veins, and did not significantly affect P, K, and B concentrations in veins. Mg-deficiency increased Fe concentration in veins of upper and middle leaves and decreased its concentration in veins of lower leaves. Under Mg-sufficiency, N, P, Mn, Zn, and B concentrations were similar among veins of upper, middle, and lower leaves; K and Mg concentrations were higher in veins of middle and lower leaves than those in veins of upper leaves; Fe concentration was higher in veins of lower leaves than those in veins of upper and middle leaves; Ca and Cu concentrations in veins increased with increasing leaf age. Under Mg-deficiency, N and Fe concentrations were higher in veins of middle leaves than those in veins of upper and lower leaves; P (Mn) concentration was higher in veins of upper and middle (lower) leaves than that in veins of lower (upper and middle) leaves; K, Ca, and Cu concentrations were higher in veins of middle and lower leaves than those in veins of upper leaves; Mg and B concentrations were similar among veins of upper, middle, and lower leaves; Zn concentration in veins increased with increasing leaf age. Interactions between Mg and leaf positions were significant only for Mg, Mn, Fe, Cu, and Zn concentrations (Figure 9).

As shown in Table S1, a significant and positive relationship was observed between blade and vein for K, Mg, Mn, and Zn concentration, but not for N, P, Ca, Fe, Cu, or B concentration.

**Figure 10:**
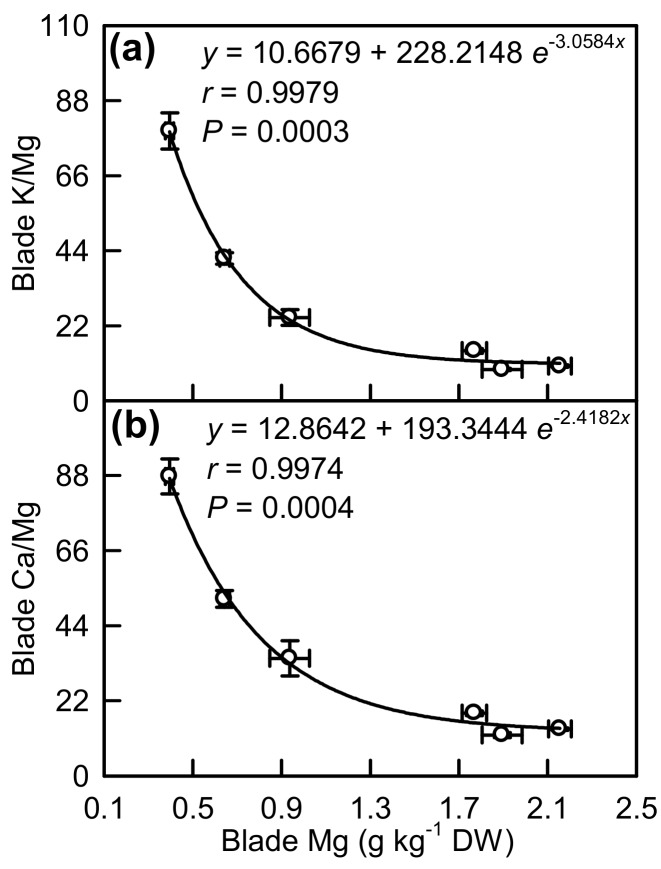
Mg concentration in relation to the K/Mg ratio (**a**) and Ca/Mg ratio (**b**) in the blades. Points represent the mean ± standard error for the independent variable (*n* = 6) and the dependent variables (*n* = 6).

As shown in Figure 10, Mg concentration was negatively related to the K/Mg and Ca/Mg ratio in the blades, respectively.

### 2.5. PCA Loading Plots

**Figure 11:**
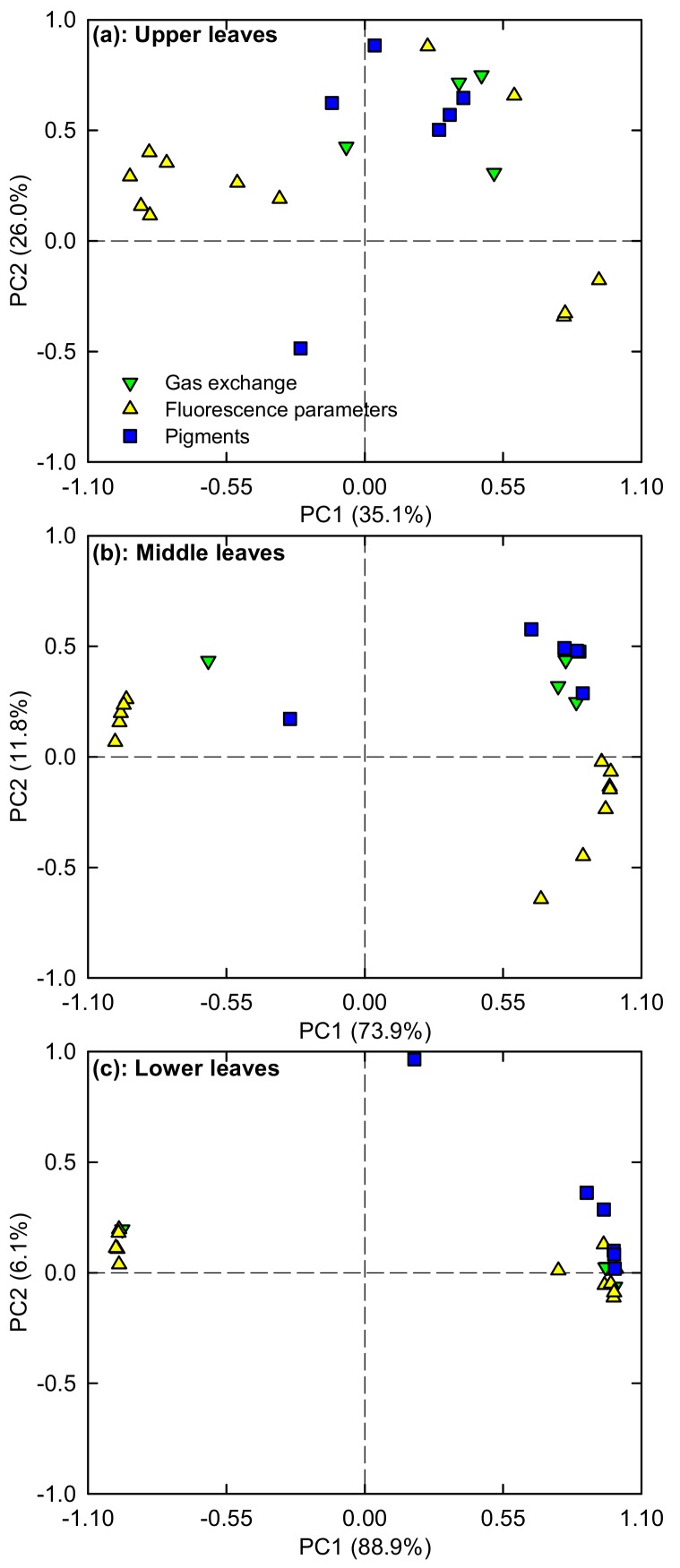
PCA loading plots of gas exchange, pigments and fluorescence parameters for upper (**a**), middle (**b**), and lower (**c**) leaves.

To determine the response patterns of photosynthesis and related physiological parameters (viz. gas exchange, pigments, and fluorescence parameters) in upper, middle, and lower leaves to Mg-deficiency, PCA was performed using these parameters (Figure 11 and Table S2). The first two components accounted for 61.1% (35.1% for PC1 and 26.0% for PC2), 85.7% (73.9% for PC1 and 11.8% for PC2), and 95.0% (88.9% for PC1 and 6.1% for PC2) of the total variation in upper, middle and lower leaves, respectively. Obviously, the contribution of PC1 and PC2 to the total variation increased with increasing leaf age.

## 3. Discussion

### 3.1. Responses of Leaf Photosynthesis and Related Parameters to Mg-Deficiency Increased with Increasing Leaf Age, and Mg-Deficiency Increased Their Differences Among Leaves with Various Ages

We found that Mg-deficiency affected photosynthesis and related parameters more in lower leaves than those in middle leaves, but it had almost no influence on them in upper leaves (Figure 2, Figure 3, Figure 5 and Figure 6). PCA indicated that the contribution of PC1 and PC2 to the total variation increased with increasing leaf age (Figure 11), suggesting that Mg-deficiency-induced alterations of these physiological parameters as a whole increased with increasing leaf age. This was also supported by our observations that Mg-deficiency symptoms gradually extended from the basal old leaves toward the top young leaves, that interveinal chlorosis appeared in Mg-deficiency middle and lower leaves, especially in the latter, and that enlargement and corkiness of midribs and main lateral veins occurred in quite a few Mg-deficiency lower leaves and a few Mg-deficiency middle leaves, but not in Mg-deficiency upper leaves (Figure 1). Mg remobilization from the old leaves to the young leaves was enhanced under Mg-starved conditions [1]. Our results indicated that Mg concentration in the blades decreased with increasing leaf age under Mg-deficiency, but it kept relatively stable under Mg-sufficiency except that Mg concentration was slightly higher in the blades of middle leaves than that in the blades of upper and lower leaves (Figure 8e). This can explain why the Mg-deficiency-induced alterations of photosynthesis and related physiological parameters increased with increasing leaf age. In addition, the differences in photosynthesis and related physiological parameters among lower, middle, and lower leaves were increased by Mg-deficiency (Figure 2, Figure 3 and Figure 6). This might be related to our data that Mg-deficiency increased the differences in Mg concentration among the blades of the lower, middle, and upper leaves (Figure 8e).

### 3.2. Antagonistic Interactions Existed between Mg and Some Elements 

The most striking Mg-deficiency effect on nutrient concentrations in the blades was the large increases in Mn and Zn concentrations (Figure 8f,i). This agrees with the studies performed in *Brassica napus* that Mg-deficiency stimulated the uptake of Mn and Zn by ~50% relative to their uptake expected from the growth rate [1], in maize that Mg-deficiency led to an increase in root and leaf Mn concentration [2], and in wheat [41] and potato [34] that Mn concentration was elevated in Mg-deficiency leaves. Our results showed that blade Mg concentration was negatively correlated with blade Mn and blade Zn concentration, respectively (Table S1), implying that an antagonistic interaction might exist between Mg and Mn (Zn). However, the antagonistic action of Mg on Mn uptake hardly existed in *Medicago sativa* [42]. It has been suggested that the competitive effects of Mg on Mn vary depending on plant species, concentrations of nutrients in the medium, and type of cultivation [2]. In potato leaves, Zn concentration did not increase in response to Mg deficiency [34]. The antagonistic effects of Mg on K and Ca uptake have been reported in *Citrus* [11,35] and onion [36]. As expected, we found that the concentrations of Ca and K were increased in Mg-deficiency the blades of upper, middle, and lower leaves with the only exception that Ca concentration did not significantly differ between Mg-deficiency and Mg-sufficiency blades of lower leaves (Figure 8c,d), as reported on *C. sinensis* leaves [33]. The competitive actions of Mg on K and Ca uptake were also supported by our findings that Mg concentration was negatively related to the K/Mg and Ca/Mg ratio in the blades, respectively (Figure 9). 

### 3.3. Mg-Deficiency-Induced Alterations of Some Nutrient Concentrations Differed between Leaf Blades and Veins, and Mg-Deficiency-Induced Enlargement and Corkiness of Veins Were Not Caused by Mg-Deficiency-Induced B-Starvation

Although K, Mg, Mn, and Zn concentration in the blades had a significant and positive relationship with the corresponding element concentration in veins, respectively (Table S1), many differences existed in Mg-deficiency-induced alterations of nutrient concentrations between the two (Figure 8; Figure 9). For examples, Mg-deficiency decreased or did not significantly alter N and P concentrations in the blades, but it increased or had no significant influence on their concentrations in veins. Mg-deficiency increased K and Ca levels in the blades except for a similar Ca level between Mg-deficiency and Mg-sufficient blades of lower leaves, but it did not affect their concentration in veins with the exception that Ca concentration was decreased in veins of Mg-deficiency upper leaves. Mg-deficiency decreased or did not alter Fe and Cu concentrations in the blades, but it increased their concentrations in veins except that Fe concentration in veins of lower leaves was decreased by Mg-deficiency.

Similar to *Citrus* B-deficiency [40], enlargement and corkiness appeared in veins of Mg-deficiency lower and middle leaves (Figure 1). However, the symptoms were not caused by Mg-deficiency-induced B-starvation, since B concentration was similar between Mg-deficiency and Mg-sufficiency veins and among veins of the lower, middle, and upper leaves (Figure 9j).

### 3.4. Impairment of Photosynthetic Electron Transport Chain Might Contribute to Mg-Deficiency-Induced Decline in Leaf CO_2_ Assimilation

The observed increased C_i_ in Mg-deficiency middle and lower leaves (Figure 3c) and the negative relationship between leaf CO_2_ assimilation and C_i_ (Figure 4b) indicated that Mg-deficiency-induced decline in leaf CO_2_ assimilation (Figure 3a) was mainly associated with non-stomatal factors, as found for longan [13], *Citrus* [3,12,43,44], and Norway spruce [45]. Peaslee and Moss suggested that Mg-deficiency inhibited maize leaf photosynthesis through Chl deterioration because chlorosis preceded the decline in photosynthesis [46]. Despite the negative relationship between Chl a + b concentration and CO_2_ assimilation in leaves (Figure 4f), the observed lower CO_2_ assimilation in Mg-deficiency middle and lower leaves (Figure 3a) might not be explained by the decreased Chl a + b level alone, since there was a far less decrease in leaf Chl a + b level than in CO_2_ assimilation (Figure 2c and Figure 3a). This is also supported by our findings showing that both DI_o_/RC and DI_o_/ABS were increased in Mg-deficiency middle and lower leaves (Figure 6g,h).

Excessive accumulation of soluble sugars (especially hexose) and starch can indirectly repress the expression of the genes encoding photosynthetic enzymes [47], and directly damage chloroplast function [48], respectively, thus inhibiting photosynthesis. The observed enlargement and corkiness of midribs and main lateral veins in Mg-deficiency middle and lower leaves indicated that Mg-deficiency impaired the structure of phloem tissue in both midribs and main lateral veins, which might block the transport of photoassimilates, thus leading the accumulation of nonstructural carbohydrates [12,33]. However, Mg-deficiency-induced inhibition of photosynthesis in middle and lower leaves could not explained alone by feedback inhibition due to the accumulation of nonstructural carbohydrates, because the concentrations of glucose, fructose, sucrose, total soluble sugars (glucose + fructose + sucrose), starch and total nonstructural carbohydrates (total soluble sugars + starch) did not significantly differ between Mg-deficiency upper and lower leaves in *C. sinensis* seedlings [3]. 

The observed positive ΔL-band in Mg-deficiency middle and lower leaves (Figure 5e) implied that the energetic connectivity (grouping) between PSII units was decreased in these leaves [21,30], especially in Mg-deficiency lower leaves. The lower grouping suggested that the PSII units of these leaves were less stable and became more fragile [31]. The positive ΔL-band has been observed in N-, P-, K-, Ca-, Mg-, S-, and Fe-deficiency maize and tomato [20], N-limited cowpea [30] and radish [49], P-starved tea [26], K-, Ca-, and Fe-deficiency maize [25], Mg- [12,43] and B-deficiency *Citrus* [19], and Fe-deficiency rapeseed [21]. 

The positive ΔK-band which occurred in Mg-deficiency middle and lower leaves (Figure 5c) indicated that OEC (especially of the Mn complex on PSII donor side) was inactivated and antennae complexes were less-connected possibly due to improper organism of membranes, thus decreasing energy transfer and absorption efficiency in these leaves, especially in Mg-deficiency lower leaves [21,50,51]. Positive ΔK-band has also been observed in all nutrient-deficiency maize and tomato [20], N-deficiency radish [49], K-, Ca-, and Fe-deficiency maize [25], Fe-deficiency rapeseed [21], B- [19] and Mg-starved *Citrus* [12,43], and P-deficiency tea [26].

We found that ABS/RC increased in Mg-deficiency middle and lower leaves, with a greater increase in the latter (Figure 6c), as found in N-deficiency radish [49], K-, Ca-, and Fe-deficiency maize and bean [25], N-, K-, Ca-, Mg-, and S-deficiency maize, N-, K-, Ca-, Mg-, and Fe-deficiency potato [20], and B-starved *Citrus* [19]. However, ABS/RC was decreased in Fe-deficiency rapeseed [21], and unaltered in P- and S-deficiency tomato, and P- and Fe-deficiency maize [20]. The observed higher ABS/RC implied that either a part of RCs was inactivated or the apparent antenna size was elevated [20,25]. The decrease in active RCs was also supported by the increased closure rate of RCs (M_o_) (Figure 6d) [49]. The inactivation of RCs is suggested to be a downregulation (adaptive) mechanism that protects the nutrient-starved leaves against photo-oxidative damage through dissipating the excess absorbed light energy [20,25]. Indeed, energy dissipation was upregulated in Mg-deficiency middle and lower leaves, as indicated by the increased DI_o_/RC and DI_o_/ABS (Figure 6g,h).

ΔJ-band, ΔI-band, and IP phase (a measure of the amount of reduced end acceptors at PSI acceptor side, the last and slowest rate-limiting step of the photosynthetic electron transport chain) are associated with the reduction of Q_A_, the reduction of plastoquinone pool, and the reduction of the electron transport acceptors in and around PSI [20,26,52]. The positive ΔJ- and ΔI-bands, and the decreased maximum amplitude of IP phase in Mg-deficiency middle and lower leaves (Figure 5c,f) suggested that Mg-deficiency increased the reduction of PSII acceptor side and the oxidation of PSI acceptor side. In other words, PSII was a more sensitive Mg-deficiency-inhibited site than PSI; Mg-deficiency affected the donor side of PSII less than the acceptor side of PSII [12,26,30,53]. This agrees with the in vitro study showing that the inactivation of PSII acceptor side was the main site for the impairment of electron transport [54]. Mg-deficiency-induced damage of PSII acceptor side was further supported by decreased F_v_ and increased F_o_, the characteristic of photoinhibitory impairment at PSII acceptor side [55]. Increased V_J_ and V_I_ and decreased maximum amplitude of IP phase have been obtained in P-starved tea [26], and B- [19] and Mg-deficiency *Citrus* [12,43].In a study, Kalaji et al. observed positive O-J and J-I normalized bands, but negative I-P normalized bands in moderate and strong Fe-deficiency rapeseed leaves, they suggested that this might be a compensatory mechanism [21]. Positive ΔJ- and ΔI-bands were observed in all nutrient-deficiency maize and tomato [20], Ca- and Fe-deficiency maize [25], and N-deficiency radish [49]. Unfortunately, the IP phase (I-P normalized band) was not provided in these studies.

Magnesium-deficiency decreased F_v_/F_m_ (a good indicator of photoinhibitory effects on PSII), ET_o_/ABS and probability that a trapped exciton moves an electron into the electron transport chain beyond Q_A_^−^ (ET_o_/TR_o_ = 1 − V_J_), increased DI_o_/RC, and altered greatly OJIP transients in middle and lower leaves (Figure 5 and Figure 6), together demonstrating that photoinhibition occurred in Mg-deficiency middle and lower leaves [21,56,57]. Nutrient deficiency-induced photoinhibitory damage to PSII has also been obtained on nutrient-deficiency *Citrus* [12,19,43], tea [26], maize, tomato [20,25], rapeseed [21], and radish [49]. The lower F_v_/F_m_ in Mg-deficiency middle and lower leaves was caused by both elevated F_o_ and reduced F_m_, especially by the former (Figure 6a,b). The elevated F_o_ might be due to accumulation of Q_A_^−^, as indicated by increased M_o_ (Figure 6d) or the impairment of OEC, as indicated by the positive ΔK-band (Figure 5e).

We observed that RE_o_/ABS (the quantum yield with which electrons reduce the PSI end-electron acceptors) and PI_abs,total_ (a measure for the performance up to reduction of PSI end-electron acceptors) were decreased in Mg-deficiency middle and lower leaves, especially in the latter (Figure 6j,l), indicating that the reduction of PSI end-electron acceptors was impaired in these leaves. Similar results have been obtained in N-deficiency radish [49], moderate and strong Fe-deficiency rapeseed [21], B- [19], and Mg-deficiency *Citrus* [12,43], Ca- and Fe-deficiency maize [25], and P-deficiency tea [26]. However, Aleksandrov et al. observed that K-, Ca-, and Fe-deficiency bean leaves had decreased RE_o_/ABS, but increased PI_abs,total_ [25]. Kalaji et al. reported that RE_o_/ABS was decreased in Fe-, K-, Mg-, and S-deficiency maize (Fe-deficiency tomato), and PI_abs,total_ was decreased in Fe-, K-, Mg-, N-, S-, and Ca-deficiency maize (Fe-, N- and S-deficiency tomato), but RE_o_/ABS and PI_abs,total_ were not significantly affected in the other nutrient-deficiency maize and tomato [20].

The decreased F_v_/F_o_ in Mg-deficiency middle and lower leaves (Figure 6f) indicated that Mg-deficiency might damage the structure of thylakoids, thus inhibiting the photosynthetic electron transport [58]. This is also supported by the previous reports that Mg-deficiency damage chloroplast ultrastructure in *Citrus* leaves [59,60]. Decreased F_v_/F_o_ has been observed on Fe-, K-, and Ca-deficiency maize, bean [25], N-deficiency radish [49], B-stressed *Citrus* [19], P-starved tea [26], Mg-deficiency *Citrus* [12,43], Mn-toxicity *Citrus* [61], low pH-stressed *Citrus* [62,63], and Al-toxicity *Citrus* [31,53,64]. The decreased F_v_/F_o_ in Mg-deficiency middle and lower leaves was due to both decreased F_v_ and increased F_o_ (Figure 6a,c,f). 

Based on these results and above discussion (Figure 6 and Figure 7), we concluded that the impairment of the whole photosynthetic electron transport chain from the donor side of PSII to the reduction of PSI end-electron acceptors (viz. structural damage to thylakoids, ungrouping of PSII units, inactivation of OEC and RCs, increased reduction of Q_A_ and plastoquinone pool at PSII acceptor side and oxidation of PSI end-electron acceptors) might be responsible for the decline in CO_2_ assimilation in Mg-deficiency middle and lower leaves.

## 4. Materials and Methods

### 4.1. Plant Culture and Mg Treatments

Plant culture and Mg treatments were made according to Cai et al. [8]. Briefly, uniform 5-week-old ‘Xuegan’ (*C. sinensis*) seedlings with single stem were transplanted to 6 L pots (two plants per pot) containing sands washed thoroughly with tap water, then cultivated in a greenhouse with a natural photoperiod at the Fujian Agriculture and Forestry University. Ten weeks after transporting, each pot was irrigated with nutrient solution (i.e., macronutrients (in mM): Ca(NO_3_)_2_, 5; K_2_SO_4_, 2; KH_2_PO_4_, 1; KNO_3_, 1; and micronutrients (in μM): Fe-EDTA, 20; H_3_BO_3_, 10; ZnSO_4_, 2; MnCl_2_, 2; CuSO_4_, 0.5, and (NH_4_)_6_Mo_7_O_24_, 0.065) at a concentration of 0 (Mg-deficiency) or 2 (control) mM Mg(NO_3_)_2_ every two days until a portion of solution leaked out from a hole in the bottom of the pot (about 500 mL). N in the nutrient solution was kept at a constant concentration by supplying equivalent moles of NH_4_NO_3_ instead of Mg(NO_3_)_2_. Sixteen weeks after treatment, upper three-quarter height, ~5-week-old), middle (two-quarter height, ~8-week-old), and lower (three-quarter height, ~11-week-old) leaves were used for all the measurements. Upper, middle, and lower leaves were fully expanded mature leaves [3].

### 4.2. Leaf Pigments and Gas Exchange

Leaf Chl a, Chl b, and Car) were determined according to Lichtenthaler after being extracted with 80 (*v/v*) acetone [65].

Leaf gas exchange was made with a CIRAS-2 portable photosynthesis system (PP Systems, Herts, UK) at a leaf temperature of 30 ± 0.2 °C, a relative humidity of 64.5 ± 0.6%, a controlled CO_2_ concentration of ~380 μmol mol^−1^, and a controlled light intensity of ~1000 μmol m^−2^ s^−1^ between 9:30 and 11:00 a.m. on a sunny day [66]. There were six replicates per treatment.

### 4.3. Leaf OJIP Transients and Related Parameters

Leaf OJIP transients were measured by a Handy PEA (Hansatech Instruments Limited, Norfolk, UK) after seedlings were dark-adapted for 3 h at room temperature. All fluorescence parameters were calculated according to Jiang et al. [31], Chen and Cheng [67], and Bank [68]. There were four replicates per treatment.

### 4.4. Assays of Elements

Lower, middle, and lower leaves were collected. Midribs and main lateral veins were carefully cut from the leaves with sharp medical scissors. A small number of blades adhered to the veins were scraped with a single knife. Then, blades (petioles, midribs, and veins removed) were collected. After being dried to a constant weight at 70 °C, all samples were ground, sieved (40-mesh), and properly stored for analysis. P was assayed colorimetrically as the blue molybdate–phosphate complexes according to Li et al. [11]. K was determined with an FP640 flame photometry (Shanghai Precision Scientific Instrument Co., Ltd, Shanghai, China). Ca and Mg were measured with a PinAAcle 900F atomic absorption spectrometer (PerkinElmer Singapore Pte Ltd, Singapore). Fe, Mn, Cu, Zn, and B were assayed with a NexION 300X inductively coupled plasma mass spectrometer (ICP-MS, PerkinElmer, Shelton, CT, USA). N was determined using an elementary Vario Max Cube Analyzer (Elementar Analysensysteme GmbH, Hanau, Germany). There were three to six replicates per treatment.

### 4.5. Statistical Analysis 

There were 20 pots (40 plants) per treatment in a completely randomized design. Results were the means ± SE (*n* = 4–6) (one plant from different pots per replicate with the exception of element analysis (four plants from two pots per replicate)) with the exception of the mean OJIP transient (only means). Data were analyzed by two-way ANOVA (three (leaf positions) × two (Mg levels)) followed by Duncan’s multiple range.

Principal component analysis (PCA) was carried out with an SPSSstatistical software (version 17.0, IBM, NY, USA) [66,69].

## 5. Conclusions

Our findings clearly demonstrated that Mg-deficiency-induced interveinal chlorosis, vein enlargement, and corkiness, and alterations of photosynthesis and related parameters were observed in middle and lower leaves, especially in the latter, but not in upper leaves. This might be related to the increased remobilization of Mg from the old leaves to the young leaves under Mg-starvation, since Mg concentration in the blades decreased with increasing leaf age. Antagonistic interactions existed between Mg and some elements. Although blade K, Mg, Mn, and Zn concentration displayed a significant and positive relationship with the corresponding vein nutrient concentration, respectively, great differences existed in Mg-deficiency-induced alterations of nutrient concentrations between leaf blades and veins. Mg-deficiency-induced enlargement and corkiness of veins were not caused by Mg-deficiency-induced B-starvation. Mg-deficiency might impair the whole photosynthetic electron transport, including structural damage to thylakoids, ungrouping of PSII units, inactivation of OEC and RCs, increased reduction of Q_A_ and plastoquinone pool at PSII acceptor side, and oxidation of PSI end-electron acceptors, thus decreasing energy transfer and absorption efficiency and the transfer of electrons to the dark reactions, and hence, the rate of CO_2_ assimilation in Mg-deficiency middle and lower leaves.

## Supplementary Materials

The following are available online at https://www.mdpi.com/2223-7747/8/10/389/s1, Table S1: Pearson correlation coefficient matrix for the concentrations of elements in leaves and leaf veins, Table S2: PCA for leaf gas exchange and related physiological parameters of upper, middle and lower leaves.
