# Supplementary material for: Magnesium-Deficiency Effects on Pigments, Photosynthesis and Photosynthetic Electron Transport of Leaves, and Nutrients of Leaf Blades and Veins in Citrus sinensis Seedlings
Source: Plants (Basel). 2019 Sep 30;8(10):389. doi: 10.3390/plants8100389 (PMC6843125; doi:10.3390/plants8100389)
Supplement: Supplementary file 1 [file plants-08-00389-s001.pdf]

Supplementary Data

Table S1. Pearson correlation coefficient matrix for the concentrations of elements in `leaf blades` and veins.

	Blades										Veins									
	**N**	**P**	**K**	**Ca**	**Mg**	**Mn**	**Fe**	**Cu**	**Zn**	**B**	**N**	**P**	**K**	**Ca**	**Mg**	**Mn**	**Fe**	**Cu**	**Zn**	**B**
Blade N	1																			
Blade P	0.8586 *	1																		
Blade K	−0.6506	−0.6790	1																	
Blade Ca	−0.3493	−0.3952	0.9314 **	1																
Blade Mg	0.6958	0.3248	−0.7099	−0.6269	1															
Blade Mn	−0.5310	−0.1342	0.5996	0.5760	−0.9683 **	1														
Blade Fe	−0.5885	−0.8150 *	0.8977 *	0.7973	−0.3791	0.2043	1													
Blade Cu	−0.1269	−0.2600	0.6522	0.6830	−0.2793	0.3051	0.5309	1												
Blade Zn	−0.6693	−0.4294	0.8559 *	0.8085	−0.9598 **	0.9092 *	0.5789	0.3973	1											
Blade B	−0.2815	−0.5137	0.5776	0.5951	−0.1494	−0.0107	0.7617	−0.0037	0.3719	1										
Vein N	−0.0576	0.1604	0.0488	0.0186	−0.2957	0.3171	−0.1357	0.3044	0.1479	−0.5317	1									
Vein P	0.4856	0.7796	−0.2173	0.0126	−0.1948	0.3628	−0.5150	0.0764	0.0836	−0.4948	0.6438	1								
Vein K	−0.3245	−0.5951	0.8685 *	0.8693 *	−0.2830	0.1756	0.9273 **	0.7610	0.5077	0.6298	−0.0411	−0.3012	1							
Leaf vein Ca	−0.1223	−0.5305	0.3966	0.3213	0.2518	−0.4018	0.6747	0.5074	−0.0950	0.3693	0.1499	−0.3988	0.7200	1						
Vein Mg	0.5383	0.0673	−0.4032	−0.3341	0.9220 **	−0.9593 **	−0.0029	−0.1311	−0.7883	0.2058	−0.4164	−0.4049	0.0668	0.5160	1					
Vein Mn	−0.6446	−0.2860	0.6715	0.6031	−0.9754	0.9836 ***	0.3056	0.3461	0.9321 **	0.0450	0.2312	0.1977	0.2484	−0.3373	−0.9331 **	1				

					***															
Vein Fe	−0.1346	−0.1023	0.5874	0.7113	−0.4820	0.3981	0.5283	0.1422	0.5993	0.6458	0.1871	0.2760	0.5161	0.2122	−0.2477	0.3321	1			
Vein Cu	−0.6886	−0.5485	0.9381 **	0.8587 *	−0.8809 *	0.8163 *	0.7034	0.6441	0.9430 **	0.3115	0.2643	0.0107	0.6843	0.1732	−0.6771	0.8625 *	0.5204	1		
Vein Zn	−0.7650	−0.5130	0.8079	0.6700	−0.9323 **	0.8754 *	0.5279	0.5280	0.9098 *	0.0889	0.4227	0.0829	0.4678	0.0411	−0.8172 *	0.9136 *	0.3869	0.9535 **	1	
Vein B	−0.1313	0.3112	0.0816	0.1467	−0.6434	0.7881	−0.3275	−0.0326	0.5314	−0.2893	0.0332	0.4628	−0.3133	−0.8501 *	−0.8126 *	0.7439	0.0038	0.3379	0.4291	1

*, **, and ***indicate significant at 5%, 1% and 1‰, respectively.

Table S2. PCA for leaf gas exchange and related physiological parameters of upper, middle and lower leaves.

Variables	Upper Leaves					Middle Leaves			Lower Leaves	
	PC1	PC2	PC3	PC4	PC5	PC1	PC2	PC3	PC1	PC2
**Leaf gas exchange**										
CO_2 assimilation	0.464	0.748	−0.296	−0.149	0.279	0.842	0.248	−0.425	0.995	−0.064
g_s	0.374	0.715	−0.537	−0.161	−0.075	0.799	0.437	−0.313	0.964	0.026
C_i	−0.073	0.426	−0.575	0.040	−0.609	−0.622	0.434	0.505	−0.963	0.195
Transpiration rate	0.514	0.307	−0.736	−0.160	−0.127	0.769	0.320	−0.290	0.957	0.025
**Chl a fluorescence parameters**										
F_o	−0.856	0.399	0.192	−0.120	0.223	−0.992	0.067	−0.065	−0.983	0.107
F_m	−0.506	0.262	0.774	−0.124	0.127	0.701	−0.644	0.201	0.769	0.010
F_v	−0.338	0.190	0.880	−0.112	0.082	0.868	−0.449	0.159	0.953	−0.056
M_o	−0.854	0.114	0.171	0.320	−0.298	−0.975	0.156	0.006	−0.977	0.037
ABS/RC	−0.889	0.157	0.130	0.267	−0.240	−0.970	0.197	−0.019	−0.977	0.197
F_v/F_o	0.791	−0.344	0.448	0.048	−0.201	0.973	−0.136	0.127	0.979	−0.050
DI_o/RC	−0.933	0.290	−0.182	0.102	0.000	−0.947	0.259	−0.063	−0.979	0.180
DI_o/ABS	−0.787	0.352	−0.434	−0.060	0.208	−0.958	0.235	−0.107	−0.989	0.112
F_v/F_m	0.796	−0.328	0.439	0.040	−0.205	0.957	−0.237	0.106	0.989	−0.113
RE_o/ABS	0.251	0.878	0.356	−0.016	0.171	0.978	−0.068	0.094	0.991	−0.089
ET_o/ABS	0.931	−0.179	0.034	−0.247	0.169	0.975	−0.148	0.047	0.950	0.127
$PI_{abs,total}$	0.594	0.656	0.434	−0.054	0.141	0.942	−0.024	0.130	0.999	0.017
**Leaf pigments**										
Chl a	0.296	0.502	−0.159	0.766	0.212	0.853	0.476	−0.003	0.990	0.100
Chl b	0.393	0.646	0.275	0.545	−0.153	0.794	0.491	−0.084	0.994	0.018
Chl a + b	0.339	0.569	−0.038	0.737	0.114	0.844	0.480	−0.019	0.992	0.082
Car	0.040	0.883	0.096	−0.429	−0.099	0.662	0.577	0.456	0.951	0.286
Chl a/b	−0.255	−0.486	−0.603	0.012	0.480	0.867	0.287	0.216	0.882	0.361
Car/Chl	−0.130	0.624	0.077	−0.743	−0.155	−0.297	0.171	0.878	0.197	0.965
**Eigen value**	7.724	5.717	4.118	2.538	1.238	16.264	2.594	1.792	19.566	1.342
**Variation percent (%)**	35.110	25.988	18.717	11.536	5.626	73.925	11.789	8.143	88.938	6.101
